# Insight into the Discriminative Efficiencies and Mechanisms of Peroxy Activation via Fe/Cu Bimetallic Catalysts for Wastewater Purification

**DOI:** 10.3390/molecules29122868

**Published:** 2024-06-16

**Authors:** Tingjin Xu, Lu Fan, Zhaokun Xiong, Bo Lai

**Affiliations:** 1 Key Laboratory of Land Resources Evaluation and Monitoring in Southwest, Ministry of Education, Sichuan Normal University, Chengdu 610068, China; 2 State Key Laboratory of Hydraulics and Mountain River Engineering, College of Architecture and Environment, Sichuan University, Chengdu 610065, China; laibo@scu.edu.cn; 3 Sino-German Centre for Water and Health Research, Sichuan University, Chengdu 610065, China

**Keywords:** Fe/Cu bimetallic catalysts, Fenton-like reactions, reactive oxygen species, sulfamethoxazole, actual wastewater experiments

## Abstract

Fe/Cu bimetallic catalysts have a synergistic effect that can effectively enhance catalytic activity, so Fe/Cu bimetallic catalysts have been extensively studied. However, the efficacy and mechanisms of Fe/Cu bimetallic catalysts’ peroxidation activation have rarely been explored. In this study, Fe/Cu bimetallic materials were fabricated to catalyze different oxidizing agents, including peroxymonosulfate (PMS), peroxydisulfate (PDS), peroxyacetic acid (PAA), and hydrogen peroxide (H_2_O_2_), for the degradation of sulfamethoxazole (SMX). The Fe/Cu/oxidant systems exhibited an excellent degradation efficiency of sulfamethoxazole (SMX). In the Fe/Cu/PMS, Fe/Cu/PDS, and Fe/Cu/PAA systems, the main reactive oxygen species (ROS) responsible for SMX degradation were hydroxyl radical (^•^OH) and singlet oxygen (^1^O_2_), while the main ROS was only ^•^OH in the H_2_O_2_ system. The differences in the surface structure of the materials before and after oxidation were examined, revealing the presence of a large amount of flocculent material on the surface of the oxidized PMS material. Anion experiments and actual body experiments also revealed that the PMS system had a strong anti-interference ability. Finally, a comprehensive comparison concluded that the PMS system was the optimal system among the four oxidation systems. Overall, this work revealed that the PMS oxidant has a better catalytic degradation of SMX compared to other oxidizers for Fe/Cu, that PMS generates more ROS, and that the PMS system has a stronger resistance to interference.

## 1. Introduction

Antiviral drugs, which are commonly used to treat various viral infectious diseases, pose a significant threat to aquatic plants and animals due to their sustained release and resistance to degradation, leading to their persistence in aquatic environments for extended periods [1]. In recent years, the increased use of pharmaceutical compounds has become a global environmental concern [2,3]. The entry of antibiotic drugs into the environment can lead to drug resistance in pathogenic bacteria and induce the development of antibiotic-resistant genes in food animals, causing severe harm to aquatic organisms and potentially endangering human health. Most antibiotics possess stable physical and chemical properties, exhibit resistance to degradation, and display prolonged half-lives. They effectively inhibit the proliferation of pathogenic microorganisms while promoting biological growth. However, their absorption by the digestive system poses challenges. Sulfamethoxazole (SMX), a medication used to treat urinary infections, is often found in water at low concentrations. SMX is considered a high-risk pollutant due to its toxicity and persistence, drawing widespread attention.

Previous studies have developed transition metal-activated sulfite generation for the production of a sulfate radical (SO_4_^•−^) to remove water pollutants [4]. Nanoscale zero-valent iron (NZVI) has been identified as an effective activator for sulfites [5]. The results of Zhou et al. also found that the NZVI/PS system exhibited higher antibiotic resistance gene removal at lower pH levels [6]. However, NZVI exhibits limitations such as instability, is prone to agglomeration, and is highly susceptible to surface passivation [7], reducing its reactivity in practical applications [8]. To address these limitations, there is an urgent need to improve NZVI. Today, bimetallic materials are being studied extensively [6], and iron-based bimetals are currently under intensive investigation as a way to enhance the reactivity of NZVI by incorporating another transition metal onto its NZVI surface [9,10]. The addition of a second transition metal can significantly accelerate the corrosion of NZVI and improve its reactivity. Copper, among other transition metals, offers advantages such as a low cost and wide applicability and can also serve as an effective activator in advanced oxidation processes (AOPs) [11]. Xu et al. found that doping with another zero-valent metal, especially nano zero-valent copper (NZVC), has been proven to promote the Fe(II)/Fe(III) cycle to active oxidants more efficiently [12]. Qin et al. also found that the reduction potential of Cu(II)/Cu(I) (0.15 V) is much lower than that of Fe(III)/Fe(II) (0.77 V), which indicates that it is thermodynamically feasible for Cu(I) to reduce Fe(III) to Fe(II) [13]. Furthermore, Xiao et al. confirmed the presence of Cu(I) and its significance for the regeneration of Fe(II) with relatively higher Cu doping [14]. Several studies have shown that the synergistic effect between iron and copper can significantly improve catalyst performance [15].

Peroxymonosulfate (PMS), peroxydisulfate (PDS), peroxyacetic acid (PAA), and hydrogen peroxide (H_2_O_2_) are widely used as oxidants in AOPs for the removal of persistent organic pollutants, disinfection, and elimination [16,17,18,19]. PMS and PDS exhibit high reactivity and stability during transport, while PAA offers advantages such as an outstanding bactericidal ability and the low generation of harmful by-products, making it suitable for wastewater disinfection [20,21]. H_2_O_2_ is extensively used for the purification of wastewater containing organic pollutants due to its versatility, high efficiency, and environmental compatibility [22,23,24].

This study employed a simple hydrothermal method to construct a highly active Fe/Cu bimetallic catalyst. Firstly, four oxidants (PMS, PDS, H_2_O_2_, PAA) were used to react with Fe/Cu to degrade SMX. A comparison of the reactive oxygen species (ROS) from the four oxidant systems was conducted to investigate the relationship between degradation efficiency and ROS as well as explore the different ROS involved in the degradation pathways. Finally, the environmental applicability of each system was analyzed based on various factors, such as the characteristics of the materials after oxidation, metal leaching, and actual water degradability. This study also revealed the efficacy and mechanism of Fe/Cu peroxide activation.

## 2. Results and Discussion

### 2.1. Characterization of the Materials

The synthesized Fe/Cu bimetal was characterized by SEM at different scales to observe the surface morphology of the material, as shown in Figures S1–S3. It can be seen that Fe^0^ formed a chain-like spherical structure with granular bumps on the surface, which were due to the attachment of Cu on the surface of Fe^0^. This result indicated the successful synthesis of the Fe/Cu bimetal.

To further investigate the surface composition of the Fe/Cu bimetal, the catalyst was characterized using XRD, and the results are shown in Figure S4. The XRD pattern shows that the fresh material exhibited a distinct diffraction peak near 2θ = 44.7°, indicating the presence of Fe^0^ (JCPDS, card No. 06-0696), but no diffraction peaks of Cu^0^ were detected at 2θ = 50.4° and 2θ = 74.1° (JCPDS, card No. 04-0836). No diffraction peaks of Cu^0^ were detected on the material’s surface after the reaction, indicating the successful synthesis of the Fe/Cu bimetallic material and the attachment of Cu^0^ to the Fe^0^ surface. The absence of a Cu^0^ diffraction peak before the reaction could be attributed to the low attachment rate of Cu^0^ on the surface layer, which may have been masked by Fe^0^ and, thus, not measured on the initial material’s surface. After the reaction, the Fe^0^ on the surface of the material was mostly consumed, revealing the presence of Cu^0^ underneath. In addition, BET tests were also conducted on the Fe/Cu bimetal, as shown in Figure S5, revealing a surface area of 2.95 m^2^/g for the Fe/Cu material. As shown in Table S1, the Fe/Cu bimetal contained 93.32% Fe and 6.62% Cu.

### 2.2. Catalytic Properties and Reproducibility of Materials

**Figure 1:**
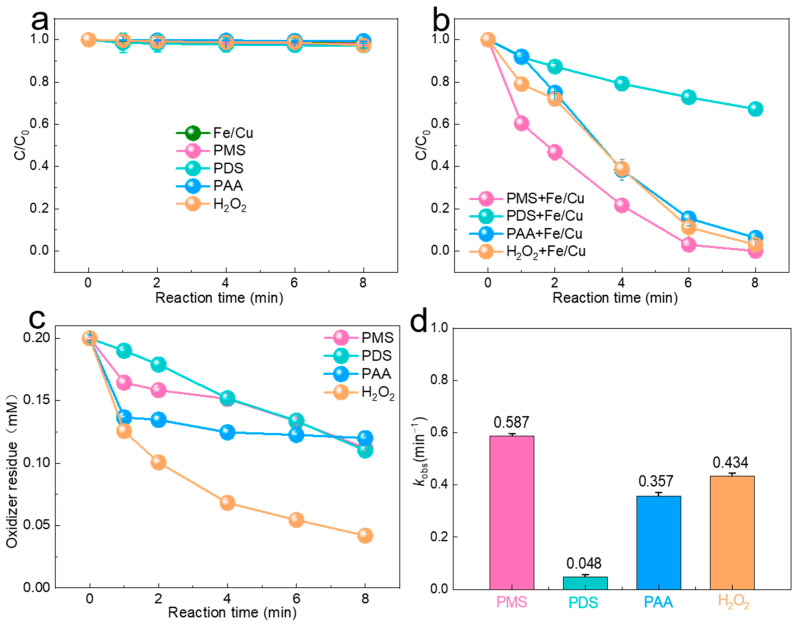
(**a**) Effect of a catalyst or oxidizer alone on SMX degradation. (**b**) SMX removal efficiency under different systems. (**c**) Oxidizer residuals for the four systems. (**d**) Kinetics of SMX degradation by different oxidizing agents. (Experiment conditions: [pollutants]_0_ = 2 mg/L, [catalyst]_0_ = 50 mg/L, [PMS]_0_ = 0.2 mM, [H_2_O_2_]_0_ = 0.2 mM, [PDS]_0_ = 0.2 mM, [PAA]_0_ = 0.2 mM, [H_2_O_2_]_0_ = 0.2 mM, and T = 30 °C).

Figure 1a shows that, when SMX is mixed with the Fe/Cu bimetal alone for 8 min, the concentration of SMX remains unchanged, indicating that the Fe/Cu bimetal alone does not absorb SMX. Similarly, when SMX is mixed with PMS, PDS, PAA, and H_2_O_2_ alone for 8 min, the SMX concentration does not change, indicating that PMS, PDS, PAA, and H_2_O_2_ alone cannot decompose to generate free radicals to react with SMX. As shown in Figure 1b, we conducted SMX degradation experiments using four oxidants at the same concentration of 0.2 mM. The PMS Fe/Cu bimetallic system demonstrated the most effective degradation, achieving 100% efficiency within 8 min, while the PDS system exhibited the lowest efficiency at 67%. The PAA and H_2_O_2_ systems showed degradation efficiencies of 94% and 97%, respectively, within the same time frame. In order to study the residual amount of oxidant in the reacted system, the residual concentrations of PMS, PDS, PAA, and H_2_O_2_ were determined using the ABTS method [25], the ion titration method, the DPD colorimetric method [26], and the titanium potassium oxalate method, respectively. Displayed in Figure 1c are the residual amounts of oxidant after 8 min of the four oxidized systems, with the residual amounts of PMS, PDS, PAA, and H_2_O_2_ measuring 0.112, 0.11, 0.12, and 0.042 mmol/L, respectively. The higher residuals of PMS, PDS, and PAA indicate a lower consumption and a higher oxidation efficiency. As shown in Figure 1d, by comparing the *k*_obs_ values of the four systems, it was found that the PMS system exhibited the strongest SMX degradation rate (0.587 min^−1^), which was higher than those of the PAA system (0.357 min^−1^) and the H_2_O_2_ system (0.434 min^−1^), as well as the PDS system (0.048 min^−1^). Overall, PMS showed the strongest SMX degradation effect compared to the other three oxidizers.

**Figure 2:**
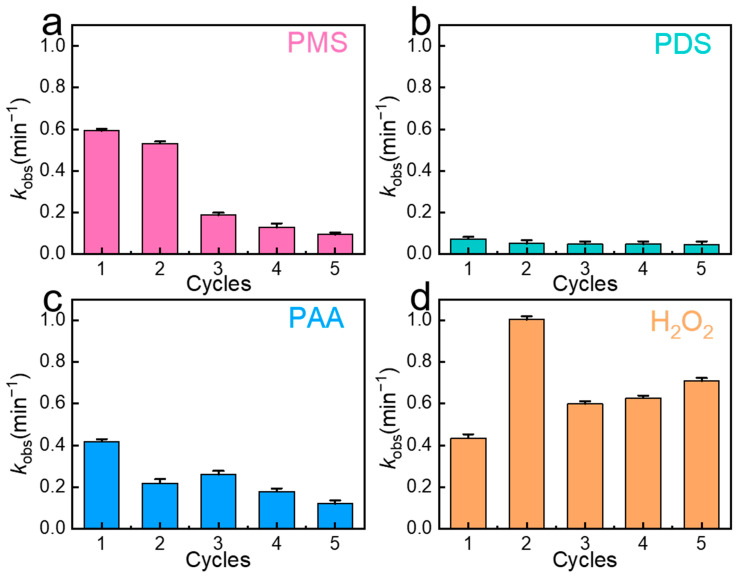
(**a**) The *k*_obs_ in reusability tests of PMS. (**b**) The *k*_obs_ in reusability tests of PDS. (**c**) The *k*_obs_ in reusability tests of PAA. (**d**) The *k*_obs_ in reusability tests of H_2_O_2_. (Experiment conditions: [pollutants]_0_ = 2 mg/L, [catalyst]_0_ = 50 mg/L, [PMS]_0_ = 0.2 mM, [H_2_O_2_]_0_ = 0.2 mM, [PDS]_0_ = 0.2 mM, [PAA]_0_ = 0.2 mM, [H_2_O_2_]_0_ = 0.2 mM, and T = 30 °C).

**Figure 3:**
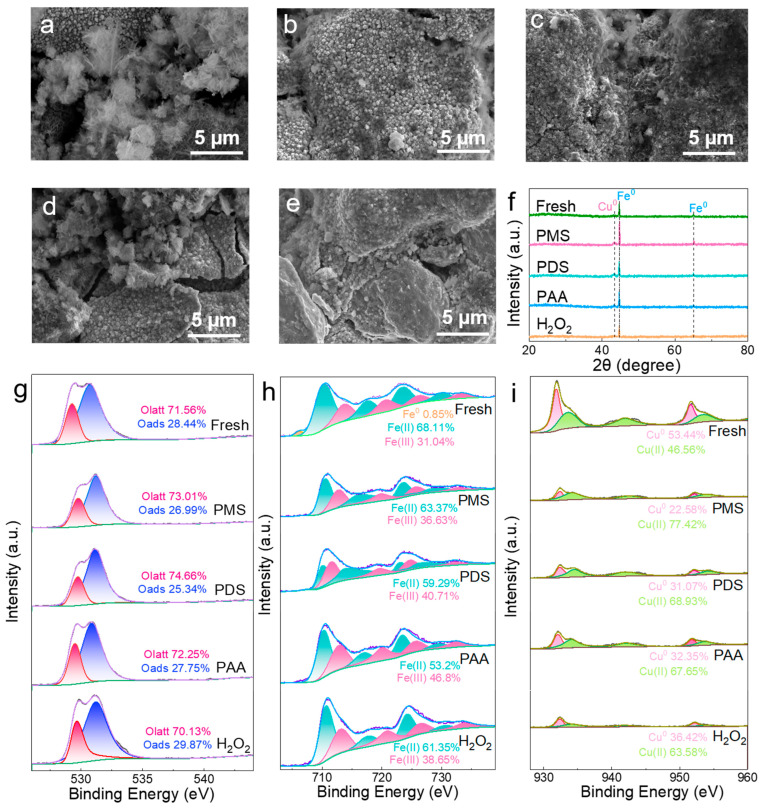
(**a**–**d**) Fe/Cu bimetal’s 5 µm SEM image after a reaction with PMS, PDS, PAA, and H_2_O_2_. (**e**) Fe/Cu bimetal’s 5 µm SEM image. (**f**) XRD patterns of fresh Fe/Cu bimetal and reaction with PMS, PDS, PAA, and H_2_O_2_. (**g**) XPS O 1s spectra of fresh and reacted Fe/Cu bimetal. (**h**) XPS Fe 2p spectra of fresh and reacted Fe/Cu bimetal. (**i**) XPS Cu 2p spectra of fresh and reacted Fe/Cu bimetal.

In order to evaluate the recyclability of Fe/Cu bimetallic materials in the four oxidation systems, five-cycle recycling tests were performed, as shown in Figure 2a–d. It was found that the degradation rate of the PMS system started to decline after two cycles, with the *k*_obs_ value decreasing to 0.186 min^−1^ in the third cycle and, further, to 0.093 min^−1^ after five cycles. This represented a decrease of 0.499 min^−1^ from the initial value of 0.592 min^−1^ after five cycles. Conversely, the degradation rate of the H_2_O_2_ system did not decay after five cycles, and the *k*_obs_ value increased from 0.434 min^−1^ to 0.709 min^−1^. The *k*_obs_ value of the PDS system changed slightly from 0.0726 min^−1^ to 0.0449 min^−1^ after five cycles, and the *k*_obs_ value of the PAA system decreased from 0.4167 min^−1^ to 0.1191 min^−1^ after five cycles. After five cycles, it was found that the H_2_O_2_ system displayed a longer cycle life, while the degradation rates of the PMS, PDS, and PAA systems showed different degrees of attenuation. Among them, the degradation rate of the PMS system showed the most pronounced decay. In order to investigate the reason for the decay of the degradation effect of the oxidant in the five-cycle recycling tests, the Fe/Cu bimetals after the reaction of the four oxidation systems were characterized by SEM, as shown in Figure 3a–e. It can be seen that the granular bumps on the surface of the Fe/Cu bimetallic material became flocculated after the reaction with the oxidant. It was presumed that the flocculent was due to the corrosion of the active sites on the surface of the Fe/Cu bimetallic material due to the reaction with the oxidant, which, in turn, impeded the subsequent reaction. In the SEM images, the flocculated material on the surface of Fe/Cu in the PMS system was the most prominent, which indicated that the Fe(II) on the surface of the Fe/Cu bimetallic material was most activated by PMS, generating more free radicals. This observation also explains why the PMS system exhibited the best degradation efficiency of SMX. The poor material lifetime of the PMS system was also due to the excessive activation of active sites, leading to the formation of a large amount of flocculated material on the material surface after multiple cycles, hindering the subsequent reactions. In the PDS system, fewer flocs were observed on the surface of the material, allowing for the retention of more active sites and enabling the material to continue reacting with PDS without significant degradation of the system efficiency after five cycles. As for the PAA and H_2_O_2_ systems, the maintenance of high activity levels after several cycles can be attributed to the lower formation of flocculated material on the material surface after the reaction, thus retaining more active sites. 

To further investigate the chemical structure changes in Fe/Cu bimetals before and after the reaction, the materials from the four oxidation systems were analyzed by XRD. As displayed in Figure 3f, it was found that the characteristic peak of Cu^0^ was enhanced after the reaction in all four systems, whereas the intensity of the characteristic peak of Fe^0^ after the reaction was weakened by the four oxidation systems. The weaker characteristic peak of Cu^0^ before the reaction was due to the lower precision of XRD, and the surface-attached Cu^0^ may not have been detected. However, it was detected after the reaction because the Fe^0^ on the surface of the Fe/Cu bimetal was consumed in large amounts by the reaction with the oxidizer, which led to a large amount of Cu^0^ being detected. The strongest characteristic peak of Cu^0^ for the PMS system could be attributed to the fact that the PMS oxidation system reacted most vigorously with the catalyst, leading to the highest consumption of Fe^0^ on the surface. The XRD results once again demonstrated the high efficiency of the PMS oxidation system.

To identify the changes in the elemental valence states on the catalyst surface during the reaction, an XPS analysis was performed on the Fe/Cu bimetal before and after the reaction. From the full XPS spectra in Figure S6, it can be seen that Fe, Cu, and O were present on the surface of the prepared Fe/Cu bimetal. In the full XPS spectra, there were two peaks close to 620 eV and 630 eV in the survey XPS spectrum for the fresh sample. These peaks were related to I3d_5/2_ and I3d_3/2_, indicating surface contamination by iodine. In Figure 3g, the high-resolution O 1s spectra can be divided into two peaks, with the peak at 530.0 eV attributed to lattice oxygen (O_2_^−^, Olatt) and the peak at 533.6 eV attributed to surface hydroxyl groups (-OH, O_ads_) [27]. After the reaction in the H_2_O_2_ system, the lattice oxygen decreased from 71.6% to 70.1%, while the surface hydroxyl groups increased from 28.4% to 29.9%. In contrast, in the other three systems, the lattice oxygen increased, and the surface hydroxyl groups decreased. This indicated that lattice oxygen was involved in the reaction in the H_2_O_2_ system [28], while surface hydroxyl groups were involved in the reaction in the remaining three systems.

The XPS spectra of Fe 2p for the Fe/Cu bimetal are shown in Figure 3h. For both Fe/Cu bimetals before and after the reaction, the Fe 2p spectra can be fitted with five peaks at 706.8 eV, 710.7 eV, 712.8 eV, 724.3 eV, and 725.8 eV, corresponding to Fe^0^ 2p_3/2_, Fe^2+^ 2p_3/2_, Fe^3+^ 2p_3/2_, Fe^2+^ 2p_1/2_, and Fe^3+^ 2p_1/2_, respectively [29]. The relatively weak Fe^0^ peak in the fresh Fe/Cu bimetal’s XPS maps may be due to the limited depth of XPS detection, as the XPS technique can only detect a depth of 5 nm on the material’s surface [30]. Since the prepared Fe/Cu bimetallic surface was coated with a layer of Cu and had an oxide film outside the Fe^0^ core, the XPS technique did not detect a strong Fe^0^ peak. However, the percentage of Fe^0^ peak of the reacted Fe/Cu bimetal decreased from 0.9% to 0, indicating that Fe^0^ was consumed in the process of degrading the pollutant. After the comparison before and after the reaction, it can be seen that the proportion of Fe (II) decreased from the initial 68.1% to 63.4%, 59.3%, 53.2%, and 61.4% after the reaction in the four systems, respectively. This indicates that Fe(II) was also consumed in the process of degrading the pollutants. Figure 3h also shows that the Fe^0^ peaks disappeared after the reaction in all four systems, indicating that Fe^0^ was oxidized in all four systems. After the reaction of the four systems, the Fe(III) peak content increased while the Fe(II) content decreased, indicating the consumption of Fe(II) during the reaction. Notably, the PMS system consumed the least amount of Fe(II), while the PAA system consumed the most Fe(II).

As shown in Figure 3i, the XPS spectra of Cu 2p of the Fe/Cu bimetal show that, both before and after the reaction, the Cu 2p spectra can be fitted with four peaks at 932.7 eV, 933.8 eV, 952.0 eV, and 953.5 eV, which correspond to Cu^0^ 2p_3/2_, Cu^2+^ 2p_3/2_, Cu^0^ 2p_1/2_, and Cu^2+^ 2p_1/2_, respectively [31]. Since the Cu2p XPS spectra are unable to distinguish between Cu^0^ and Cu^+^ because of their significant overlap, only Cu^0^ is discussed in this article. The peaks of Cu^0^ and Cu(II) are more pronounced in a fresh Fe/Cu bimetal, while the peak intensities of both Cu^0^ and Cu(II) are significantly weakened after the reaction. The results indicated that the Cu^0^ content in all four systems decreased significantly from the initial 53.4% to 22.6%, 31.1%, 32.4%, and 36.4%, respectively, at the end of the reaction. This suggests that Cu^0^ was consumed during the process of the reaction. Notably, the H_2_O_2_ system showed the least reduction in Cu^0^ substance content, while the PMS system showed the most reduction. This supported the effectiveness of the H_2_O_2_ system in cycling experiments and the ineffectiveness of the PMS system. In order to investigate whether the catalytic activity was due to the release of copper from the solution (homogeneous reaction) or due to heterogeneous Fe/Cu catalysts, the metal content of the solution after the reaction was determined (Figure S7). Subsequently, homogeneous experiments with the same concentration of copper ions were carried out, and the results are shown in Figure S8, indicating that the catalytic activity of the material is indeed due to the heterogeneous iron/copper catalyst.

The above results explored the material surface before and after the Fe/Cu bimetallic reaction in different systems. However, since the degradation of SMX is related to the generation of ROS within a system, the ROS generation in the four systems was investigated next.

### 2.3. Identification of Reactive Oxygen Species (ROS)

**Figure 4:**
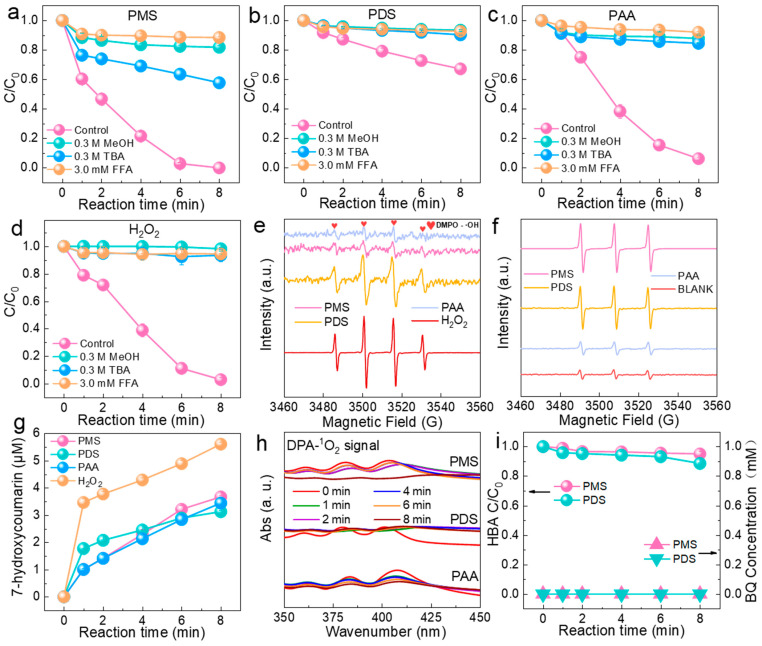
(**a**–**d**) The effects of different scavengers on SMX degradation in PMS/PDS/PAA/H_2_O_2_ systems. (**e**) DMPO-trapped EPR spectra of PMS/PDS/PAA/H_2_O_2_ systems. (**f**) TEMP-trapped EPR spectra of PMS/PDS/PAA systems. (**g**) Concentrations of 7-hydroxycoumarin produced by the PMS/PDS/PAA/H_2_O_2_ systems. (**h**) UV spectra of DPA degradation in PMS/PDS/PAA/H_2_O_2_ systems. (**i**) Variation in HBA and BQ concentration in PMS/PDS systems. (Experimental conditions: [TBA]_0_ = 0.3 M, [MeOH]_0_ = 0.3 M, [FFA]_0_ = 3 mM, [DPA]_0_ = 1 g/L, and [HBA]_0_ = 5 g/L).

It has been demonstrated that several ROS can be generated during the Fenton-like oxidation process (e.g., ^•^OH, SO_4_^•−^, etc.) [32]. Therefore, to investigate the major ROS of different systems, the results of this study were analyzed by quenching experiments, EPR analyses, and chemical probe experiments in all four systems. As shown in Figure 4a–d, MeOH (*k*(^•^OH_,_ MeOH) = 9.7 × 10^8^ M^−1^ s^−1^, *k*(SO_4_^•−^, MeOH)= 2.5 × 10^7^ M^−1^ s^−1^), TBA (^•^OH_,_ TBA) = 6.6 × 10^8^ M^−1^ s^−1^, *k*(SO_4_^•−^, TBA) = 7.7 × 10^7^ M^−1^ s^−1^), and FFA (*k*(^1^O_2_, FFA) = 1.2 × 10^8^ M^−1^ s^−1^) were used as particular scavengers for ROS within different systems [33,34]. In the PMS oxidation system, the degradation ratio of SMX within the reaction time decreased from 100% to 18.2%, 42.3%, and 11.5% after the addition of MeOH, TBA, and FFA, respectively. Compared with TBA, the addition of MeOH showed stronger inhibition on SMX degradation, which indicated the presence of both ^•^OH and SO_4_^•−^ in the system, the similar quenching effect of FFA with TBA indicated that there was little ^1^O_2_ generated within the PMS oxidation system. The degradation ratio of SMX decreased from 32.8% to 6.7% after adding MeOH to the PDS oxidation system, and it decreased to 9.7% after adding TBA and 7.3% after adding FFA. The inhibition effects after adding MeOH and TBA were comparable, which indicated that only ^•^OH was contained in the system, while the similar quenching effects of FFA with TBA indicated that there was little ^1^O_2_ generated within the PDS oxidation system. The degradation ratio of SMX in the PAA oxidation system decreased from 37.1% to 12.2% after adding MeOH, decreased to 15.5% after adding TBA, and decreased to 8% after adding FFA. The same degree of inhibition after adding MeOH and TBA indicated that only ^•^OH was contained in the system, whereas the similar quenching effect of FFA with TBA indicated that there was little ^1^O_2_ generated within the PAA oxidation system. The degradation ratio of SMX in the H_2_O_2_ oxidation system decreased from 97% to 1.7% after adding MeOH, 6.4% after adding TBA, and 8.7% after adding FFA, and then it decreased to 5.5%. The same inhibitory effect after adding MeOH and TBA indicated that the system contained only ^•^OH, while the similar quenching effect of FFA with TBA indicated that there was little ^1^O_2_ generated within the H_2_O_2_ oxidation system.

Moreover, to further explore the generation of ROS within different oxidation systems, EPR experiments were carried out using DMPO and TEMP as the spin-trapping agents. The results in Figure 4e–f illustrate that the EPR signals of DMPO-SO_4_^•−^ cannot not be observed in the PMS and PDS systems, and, combined with the results of the quenching experiments, the DMPO-SO_4_^•−^ signal is not detected in the PMS system due to its low production of SO_4_^•−^, whereas there is no SO_4_^•−^ generated in the PDS system. Moreover, the 1:2:2:1 quadruple signal for DMPO-^•^OH was detected in all PMS, PDS, PAA, and H_2_O_2_ systems, which demonstrated the generation of ^•^OH in the above four systems. The PMS, PDS, and PAA systems detected the strong 1:1:1 triplet signal for the characteristic peaks of ^1^O_2_, indicating that ^1^O_2_ was produced in three of the four systems, with the exception of the H_2_O_2_ system.

Chemical probe experiments were conducted to further explore the existence of ^•^OH in the four systems. As shown in Figure 4g, this study utilized the reaction of coumarin and ^•^OH to generate 7-hydroxycoumarin for the quantitative determination of ^•^OH for the four oxidation systems [35]. As shown above, the ^•^OH production was 3.67 μM for the PMS system, 3.13 μM for the PDS system, 3.45 μM for the PAA system, and 5.61 μM for the H_2_O_2_ system. The H_2_O_2_ system produced the most ^•^OH, while the PDS system had the lowest ^•^OH production.

Next, we analyzed the role played by ^1^O_2_ in the oxidative degradation of pollutants, and used 9,10-diphenylanthraquinone dyes (DPA)’s oxidation to analyze the generation of ^1^O_2_ [36]. As Figure 4h shows, the intensity of the absorption peak near 378 nm gradually diminished during the first 4 min in the PMS system, which indicated the continuous production of ^1^O_2_. However, the peak intensity at 6 min was stronger than that at 4 min, suggesting a decrease in the accumulation of ^1^O_2_ due to the depletion of PMS. In the PDS system, however, the intensity of the absorption peak near 378 nm weakened rapidly in the first 1 min, which indicated the rapid production of ^1^O_2_ within the beginning of the reaction, and the increase of 378 nm after 2 min indicated the decrease in the accumulation of ^1^O_2_. In the PAA system, the intensity of the absorption peak near 378 nm decreased rapidly within 1 min, which indicated the rapid production of ^1^O_2_, and then the intensity of the absorption peak near 378 nm gradually decreased in a flat manner after 2 min, which indicated the slow production of ^1^O_2_. Overall, the intensity of the absorption peak near 378 nm in the PDS system and the PAA system decreased more slowly than in the PMS system. The results ulteriorly suggested that the PMS system produced the most ^1^O_2_.

Further probe experiments were performed on the detection of SO_4_^•−^. The reaction was carried out by replacing SMX in the original system with 5 g/L p-hydroxybenzoic acid (HBA), as shown in Figure 4i. The concentration of HBA was almost unchanged after 8 min, and the product benzoquinone was not measured in the system, which indicated that SO_4_^•−^ production was weak or did not generate production in the PMS and PDS systems. Concerning SO_4_^•−^, summarizing the above experimental findings, it can be found that trace amounts of SO_4_^•−^ were produced in the PMS system, and SO_4_^•−^ was not a major active species in the PMS system, while no SO_4_^•−^ was produced in the PDS system.

To study the active species of the system more comprehensively, probe experiments were conducted to clarify whether Fe (IV) and O_2_^•−^ were produced in the four systems. PMSO is typically employed as a probe to identify high-valent iron species [36]. In this way, 10 µM PMSO was used to measure the consumption of PMSO and the production of PMSO_2_ to determine whether Fe (IV) was produced in the four systems, as shown in Figure S9. Although there were minor fluctuations in the PMSO concentration over 8 min, the concentration was essentially unchanged, and no production of PMSO_2_ was detected. This indicated that the system did not produce Fe (IV). Finally, superoxide dismutase (SOD) also quenched the O_2_^•−^ of the four systems, as shown in Figure S10, and it was found that the addition of SOD did not inhibit any of the four oxidized systems, indicating that O_2_^•−^ was not generated.

In conclusion, ^•^OH and ^1^O_2_ were the major active species in the PMS, PDS, and PAA systems, while the active species in the H_2_O_2_ system was only ^•^OH. And, the PMS system produced the most ^1^O_2_ as well as substantial ^•^OH in the four systems. This may be the reason for the superiority of the PMS system in degrading SMX.

### 2.4. Effects of Co-Existing Substances

**Figure 5:**
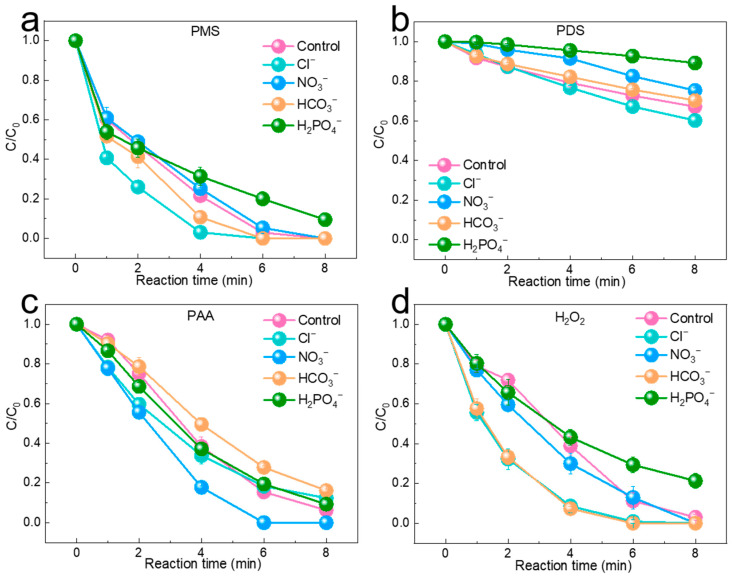
(**a**–**d**) The effects of Cl^−^, NO_3_^−^, HCO_3_^−^, and H_2_PO_4_^−^ on SMX degradation in the PMS/PDS/PAA/H_2_O_2_ systems. (Experimental conditions: [pollutants]_0_ = 2 mg/L, [catalyst]_0_ = 50 mg/L, [PMS]_0_ = 0.2 mM, [H_2_O_2_]_0_ = 0.2 mM, [PDS]_0_ = 0.2 mM, [PAA]_0_ = 0.2 mM, [H_2_O_2_]_0_ = 0.2 mM, and [Cl^−^]_0_ = [NO_3_^−^]_0_ = [HCO_3_^−^]_0_ = [H_2_PO_4_^−^]_0_ = 5 mM).

In real water bodies, inorganic ions can affect the performance of catalysts by influencing the decomposition of oxidant molecules and the acquisition of free radicals. Among these inorganic anions, Cl^−^, NO_3_^−^, HCO_3_^−^, and H_2_PO_4_^−^ are commonly found in most aqueous environments. Therefore, the effect of anions was further evaluated by adding them to the four oxidation systems. As shown in Figure 5a–d, the degradation of SMX was accelerated due to the reaction of Cl^−^ with ^•^OH to form ClOH^•−^ under acidic conditions, and ClOH^•−^ further reacted with H^+^ to form Cl^−^ [37]. Thus, the addition of Cl^−^ in all four oxidation systems accelerated the degradation of SMX. The inhibition of H_2_PO_4_^−^ may have been attributed to the presence of buffer ions, which affected the pH of the reaction system, thereby impacting the activation effect. HCO_3_^−^ promoted degradation in the PMS and H_2_O_2_ systems but inhibited degradation in the PDS and PAA systems. NO_3_^−^ played a promoting role in the PAA system, while it showed slight inhibition in the other systems. Specifically, in the PMS system, the degradation rate declined to 91% except for H_2_PO_4_^−^, while it remained at 100% in the presence of other ions. Similarly, the PDS system failed to achieve a high degradation effect, and the degradation rate in the PAA system reached 100% only in the presence of NO_3_^−^ ions, while the degradation rate was inhibited in other ions. In the H_2_O_2_ system, the degradation rate decreased to 79% under the influence of H_2_PO_4_^−^, while it remained 100% in the presence of other anions. In general, among the four systems, the PMS system showed the strongest environmental applicability as well as anti-interference ability.

**Figure 6:**
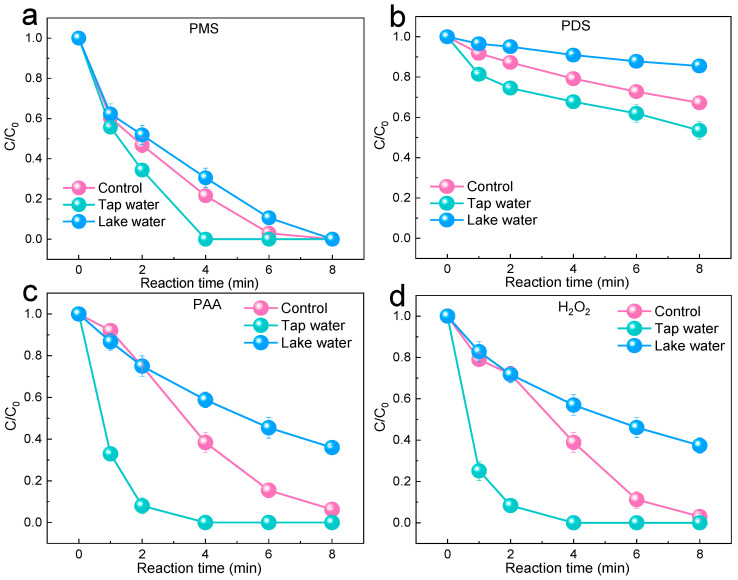
(**a**–**d**) The effects of tap water and lake water on SMX degradation in the PMS/PDS/PAA/H_2_O_2_ systems. (Experimental conditions: [pollutants]_0_ = 2 mg/L, [catalyst]_0_ = 50 mg/L, [PMS]_0_ = 0.2 mM, [H_2_O_2_]_0_ = 0.2 mM, [PDS]_0_ = 0.2 mM, [PAA]_0_ = 0.2 mM, and [H_2_O_2_]_0_ = 0.2 mM).

The investigation also explored the degradation effect of SMX using the four oxidation systems in tap water and actual lake water, as shown in Figure 6a–d. The higher degradation activity observed in tap water could be attributed to the promotion of SMX degradation by the presence of Cl^−^ in tap water. Conversely, the inhibitory effect observed in actual lake water was due to the high organic matter content in the lake water. However, the PMS system was able to achieve 100% degradation in real lake water within 8 min. Compared to other oxidation systems, the PMS system maintained a strong degradation effect in both lake water and tap water. Therefore, among the four oxidation systems, the PMS system demonstrated the best practicality. 

### 2.5. Possible Degradation Pathway

**Figure 7:**
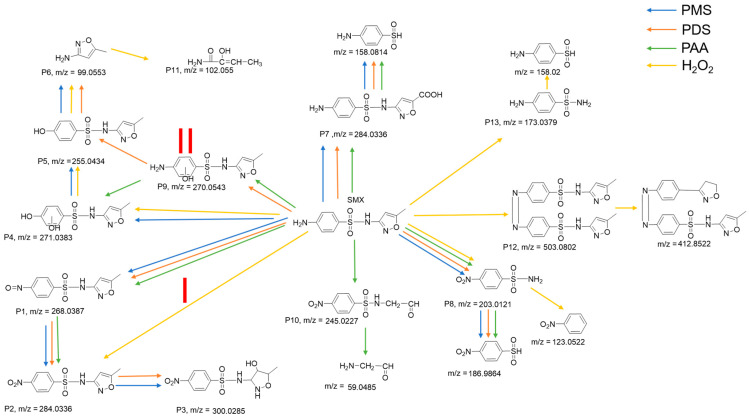
The proposed degradation pathways of SMX in Fe/Cu/oxidant systems.

The intermediates of SMX in the four oxidation systems were obtained by UPLC-QTOF-MS/MS. A total of 13 intermediates were detected during the oxidative removal of SMX by the Fe/Cu bimetallic system, based on the exact mass of the transformation products and their fragments. These were named from P1 to P13 according to their *m*/*z* values, and the corresponding structures and other details are provided in Table S2. The possible SMX degradation pathways are shown in Figure 7, summarizing the multiple degradation pathways in the four oxidation systems. In process I, P1 and P2 were obtained from the oxidation of the amino group on the benzene ring of SMX to nitro, and the hydroxyl substitution on the heterocyclic ring led to the formation of P3. This process occurred in both the PMS and PDS systems, whereas P3 was not detected in the PAA system, and only P2 was detected in the H_2_O_2_ system. In process II, the first two steps involved direct hydroxyl substitution and hydroxyl substitution on the benzene ring of SMX in the aromatic portion. This occurred in both the PMS and H_2_O_2_ systems. In the PMS and H_2_O_2_ systems, SMX first underwent a direct hydroxyl substitution on the aromatic part, and the amino group on the benzene ring was hydroxylated to form P4 and P5 [38]. The S-N bond of P5 may have undergone δ-cleavage to produce P6 [39], and P11 was generated by the ring opening of P6, observed in the H_2_O_2_ system. In contrast, in the PAA system, the generation of P4 was preceded by the direct hydroxyl substitution on the benzene ring to form P9 from SMX. In the PDS system, the generation of P9 from PDS was preceded by the direct hydroxyl substitution on the benzene ring and the deletion of benzene from P4. In other processes, the PAA system also generated P10, formed by oxidizing the amine group on the benzene ring of SMX first to nitro and then oxidizing to open the ring. The dimer of SMX P12 was found in the H_2_O_2_ system, as well as P13, obtained by breaking the C-N bond to remove the heterocyclic ring from SMX. Moreover, the amino group on the benzene ring of SMX was oxidized, and, then, the C-N bond was broken to remove the heterocyclic ring, resulting in P8, observed in all four systems. Additionally, P7, obtained by the direct oxidation of the methyl group on the heterocycle of SMX to a carboxyl group, was found in all three systems—PMS [40], PDS, and PAA.

## 3. Materials and Methods

### 3.1. Materials

The chemical reagents utilized in this study included trace amounts of zero-valent iron (Fe^0^) powder, sodium sulfate (Na_2_SO_4_), copper sulfate pentahydrate (CuSO_4_•5H_2_O), disodium ethylenediaminetetraacetate (EDTA-2Na), sodium hydroxide (NaOH), sulfuric acid (H_2_SO_4_), sulfamethoxazole (SMX), sodium thiosulfate (Na_2_S_2_O_3_-5H_2_O), deionized water, peroxymonosulfate (PMS), peroxodisulfate (PDS), peroxyacetic acid (PAA), hydrogen peroxide (H_2_O_2_), NaCl, NaNO_3_, NaHCO_3_, NaH_2_PO_4_, C_9_H_6_O_2_, methyl alcohol (MeOH), methyl phenyl sulfoxide (PMSO), tert-butanol (TBA), furfuryl alcohol (FFA), 5,5-dimethyl-1-pyrroline N-oxide (DMPO), 2,2,6,6-tetramethyl-4-piperidinyloxyl (TEMP), and 9,10-diphenylanthraquinone dyes (DPA) purchased from Aladdin (Shanghai, China). All the chemical reagents employed were of analytical grade and used directly without further purification. Deionized water was used throughout the experiment.

### 3.2. Catalyst Preparation

A 200 mL volume of a mixed solution of 3.894 g/L CuSO_4_•5H_2_O and 0.580 g/L EDTA-2Na•2H_2_O complexing agent (with a molar ratio of Cu/EDTA of 1/10) was supplemented with 3 g of Fe^0^ (with a theoretical Cu mass loading of 0.066 g Cu/g Fe), which was then mixed for 4 min at 40 °C by a mechanical stirrer (350 rpm). After 2 min of precipitation process, the prepared Fe/Cu particles were separated from the supernatant. Subsequently, the separated Fe/Cu particles were rinsed three times with deionized water. Finally, the obtained Fe/Cu particles were baked at 60 °C for 4 h.

### 3.3. Experimental Procedure

All the experiments were carried out in a 250 mL glass beaker containing 200 mL of SMX (2 mg/L) with a constant stirring rate of 300 rpm at 30 ± 1 °C. Each reaction was initiated with the required dosages of catalysts and oxidants. At each time interval, 0.22 µm polytetrafluoroethylene syringe filter discs were used to filter the samples, which were then quenched by a Na_2_S_2_O_3_ solution (50 μL) before analysis. For the recycling tests, the catalysts were separated and washed with deionized water several times. The initial pH was adjusted by H_2_SO_4_ or NaOH after the addition of the oxidants. All the experiments were carried out in duplicate or triplicate, and the obtained data were averaged.

### 3.4. Analytical Methods

The structure and morphology of the samples were confirmed by X-ray diffraction (XRD, Shimadzu, Kyoto, Japan, XRD-7000), scanning electron microscopy (SEM, SU-8010, Hitachi, Tokyo, Japan), and transmission electron microscopy (TEM, FEI Talos F200x, FEI Company, Waltham, MA, USA). X-ray photoelectron spectroscopy (XPS, AXIS Ultra DLD, Kratos Co., Kyoto, Japan) was used to analyze the elemental composition and chemical oxidation state. The nitrogen adsorption curves were tested by the ASAP 2420 micromeritic instrument, and the Brunauer–Emmett–Teller surface area was analyzed by the Brunauer–Emmett–Teller model. Electron paramagnetic resonance (EPR) spectra were obtained on a Bruker EMX plus X-band CW EPR spectrometer (Bruker, Billerica, MA, USA) (microwave frequency, 9.83 GHz; microwave power, 2.00 MW), with the parameters detailed in Text S1.

## 4. Conclusions

A comprehensive study was conducted to investigate the degradation efficiency, active species, and degradation pathways of Fe/Cu bimetallic materials in the PMS, PDS, PAA, and H_2_O_2_ systems. The surface morphology of the Fe/Cu bimetallic materials before and after the reactions in each system was observed to select the optimal degradation system. The results indicated that the PMS system exhibited the highest degradation efficiency among the four systems. In the PMS, PDS, and PAA systems, the primary active species responsible for SMX degradation were ^•^OH and ^1^O_2_, while, in the H_2_O_2_ system, the predominant active species was only ^•^OH. Flocculent substances were found on the material surfaces after the reaction in all the systems, with the highest amount observed in the PMS system, demonstrating the highest oxidation efficiency. Additionally, the XPS comparison revealed that the H_2_O_2_ system exhibited the most vigorous oxidation. Findings from anion and actual water body experiments further demonstrated that the PMS system possessed the strongest anti-interference capability among the four oxidation systems. Consequently, the PMS system was the optimal choice among the four systems.

## Data Availability

The original contributions presented in the study are included in the article/Supplementary Materials, further inquiries can be directed to the corresponding authors.

## Supplementary Materials

The following supporting information can be downloaded at https://www.mdpi.com/article/10.3390/molecules29122868/s1, Text S1: Details of analytical methods; Figure S1: SEM image of Fe-Cu bimetallic catalysts; Figure S2: SEM image of Fe-Cu bimetallic catalyst; Figure S3. SEM image of Fe-Cu bimetallic material; Figure S4: The XRD patterns of the Fe-Cu bimetallic catalyst; Figure S5: N_2_ adsorption isotherms of the Fe-Cu bimetal; Figure S6: XPS full spectra of fresh and reacted Fe/Cu bimetal; Figure S7: Dissolution of iron and copper ions in different systems; Figure S8: Cu ion homogeneous phase experiment; Figure S9: PMSO degradation and PMSO_2_ generation in PMS/PDS/PAA/H_2_O_2_ systems; Figure S10: Degradation of SMX in four systems after the addition of SOD; Figure S11: Fragment ions’ identification of SMX; Figure S12: Fragment ions’ identification of P1; Figure S13: Fragment ions’ identification of P2; Figure S14: Fragment ions’ identification of P3; Figure S15: Fragment ions’ identification of P4; Figure S16: Fragment ions’ identification of P5; Figure S17: Fragment ions’ identification of P6; Figure S18: Fragment ions’ identification of P7; Figure S19: Fragment ions’ identification of P8; Figure S20: Fragment ions’ identification of P9; Figure S21: Fragment ions’ identification of P10; Figure S22: Fragment ions’ identification of P11; Figure S23: Fragment ions’ identification of P12; Figure S24: Fragment ions’ identification of P13; Table S1: The ICP-OES of the Fe-Cu bimetal; Table S2: Transformation intermediates of SMX detected by UPLC-QTOF-MS/MS.
