# Supplementary material for: Insight into the Discriminative Efficiencies and Mechanisms of Peroxy Activation via Fe/Cu Bimetallic Catalysts for Wastewater Purification
Source: Molecules. 2024 Jun 16;29(12):2868. doi: 10.3390/molecules29122868 (PMC11206741; doi:10.3390/molecules29122868)
Supplement: Supplementary file 1 [file molecules-29-02868-s001.zip › molecules-3010015-supplementary.pdf]

*Supporting information for*

**Insight into the Discriminative Efficiencies and Mechanisms of Peroxy Activation via Fe/Cu Bimetallic Catalysts for Wastewater Purification**

**Tingjin Xu [1], Lu Fan [1,*], Zhaokun Xiong [2,3,*] and Bo Lai [2,3]**

1 Key Laboratory of Land Resources Evaluation and Monitoring in Southwest, Ministry of Education, Sichuan Normal University, Chengdu, 610068, China
2 State Key Laboratory of Hydraulics and Mountain River Engineering, College of Architecture and Environment, Sichuan University, Chengdu 610065, China; laibo@scu.edu.cn
3 Sino-German Centre for Water and Health Research, Sichuan University, Chengdu 610065, China
* Correspondence: fanlu@sicnu.edu.cn (L.F.); scuxzk@scu.edu.cn (Z.X.)

**Text S1. Details of analytical methods**

An X-ray photoelectron spectrometer (ThermoFischer, ESCALAB Xi+, USA) was used for this experiment. In particular, the vacuum of the analyzing chamber was 8xl0-10 Pa, the excitation source was Al ka ray (hv=1486.6 eV), the operating voltage was 12.5 kV, the filament current was 16 mA, and the signal was accumulated for 10 cycles. Passing-Energy was tested at 100ev for the full spectrum and 30eV for the narrow spectrum, with a step size of 0.1eV, a dwell time of 40-50ms, and a charge correction with C1s=284.80eV binding energy as the energy standard. Spot 650 microns. The X-ray diffraction patterns were measured with Shimadzu XRD-7000 instrument in reflection mode with Cu K$\alpha$ radiation ($\lambda$ = 1.542 Å, 40 kV and 30 mV) at a scanning rate 5° min$^{-1}$ from 5° to 80° (2θ). The morphology of as-prepared samples was examined by scanning electron microscope (SEM, SU-8010, Hitachi) and transmission electron microscopy (TEM, FEI Talos F200x, FEI Company, USA).

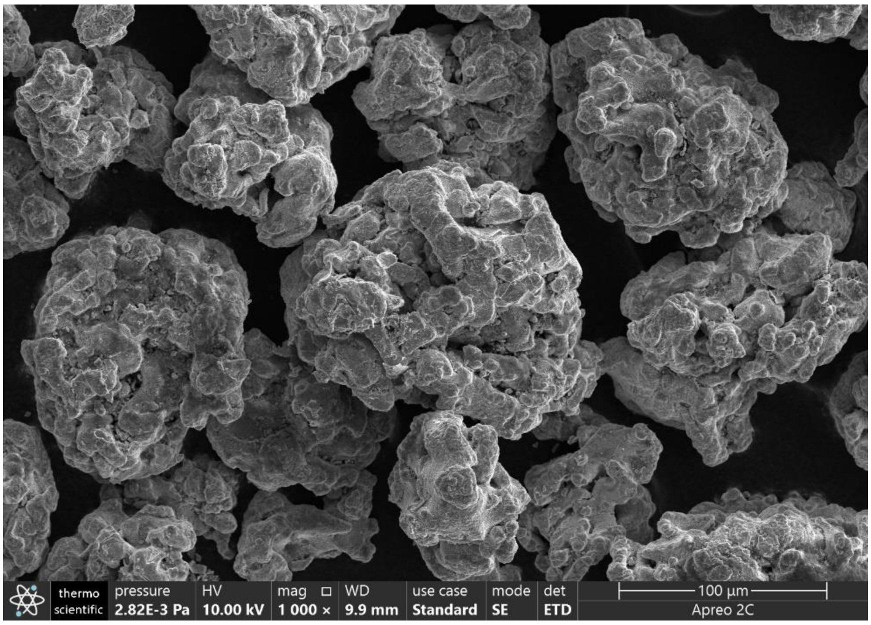

**Figure S1.** SEM image of Fe-Cu bimetallic catalysts.

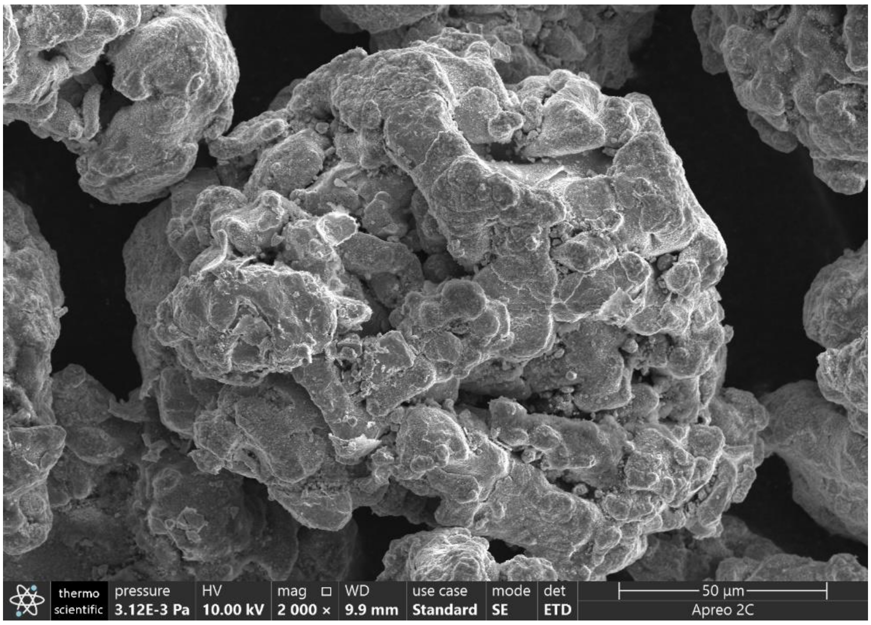


**Figure S2.** SEM image of Fe-Cu bimetallic catalyst.

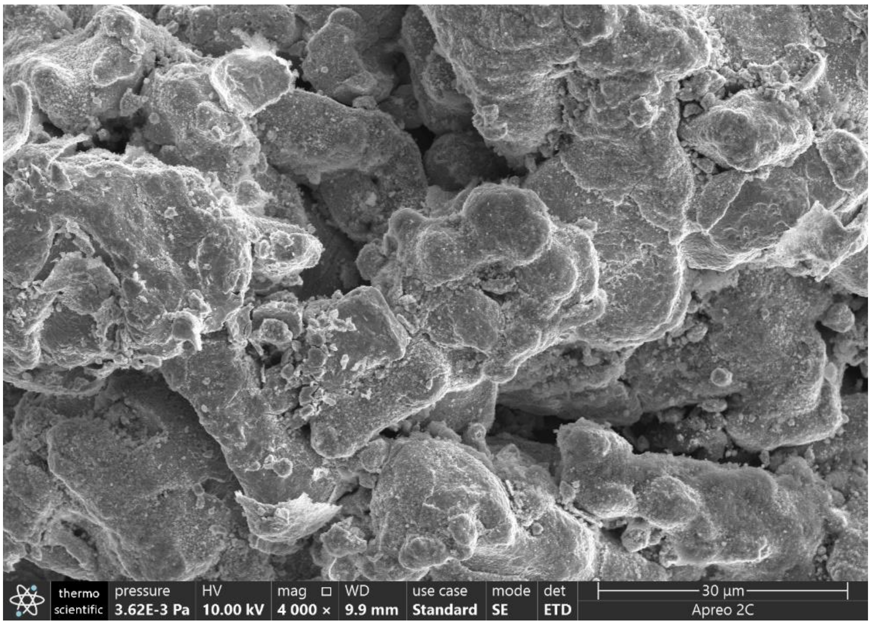


**Figure S3.** SEM image of Fe-Cu bimetallic material.

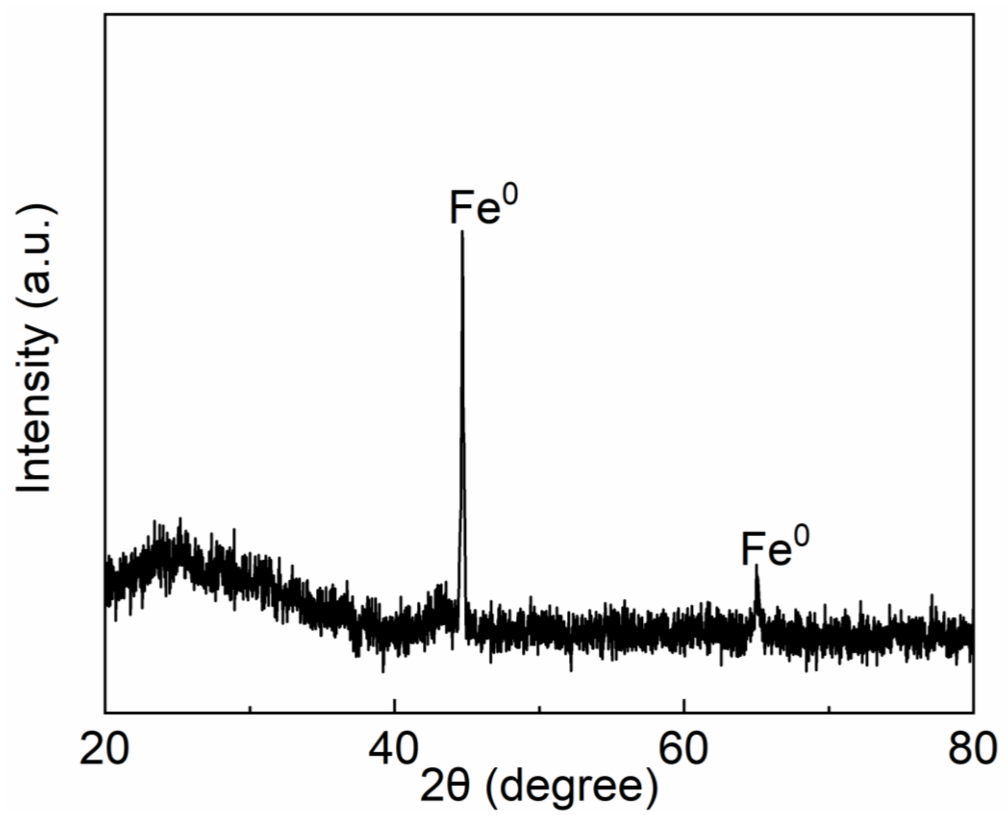


**Figure S4.** The XRD patterns of Fe-Cu bimetallic catalyst.

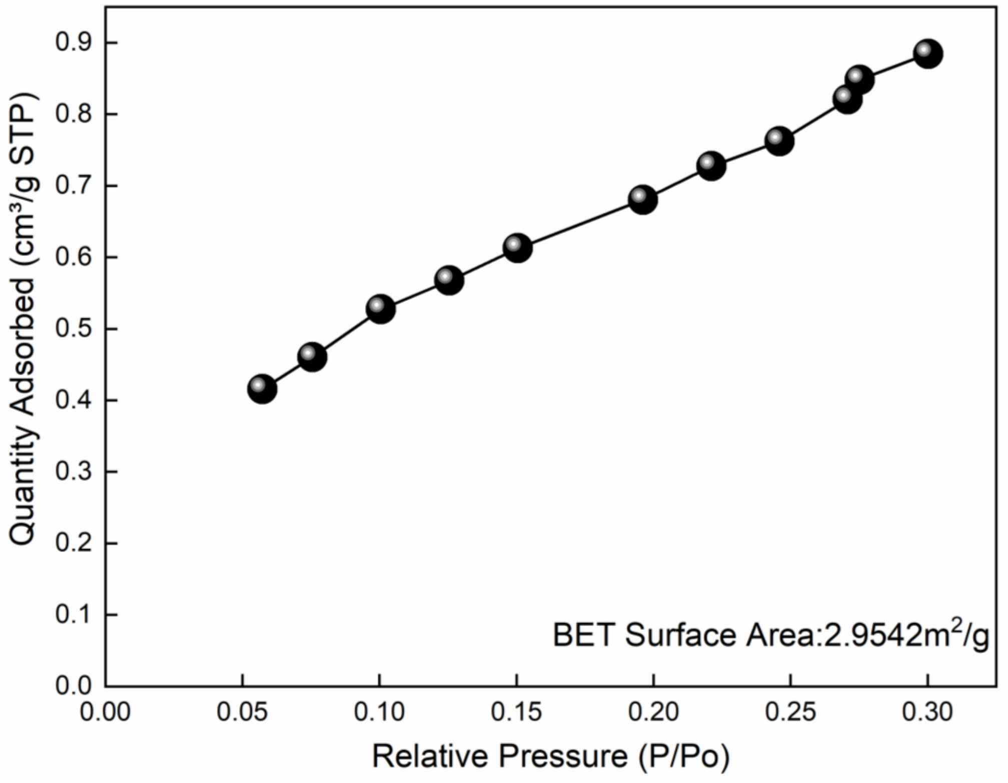


**Figure S5.** $N_2$ adsorption isotherms of the Fe-Cu bimetal.

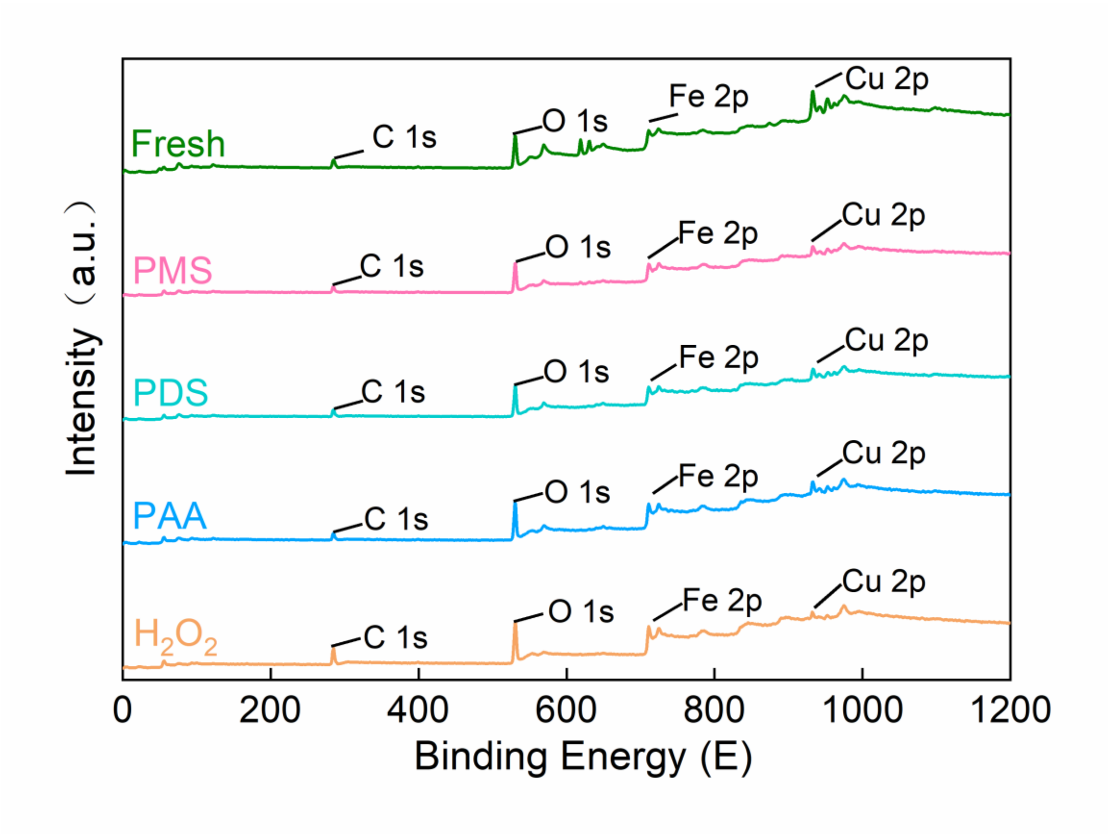


**Figure S6.** XPS full spectra of fresh and reacted Fe/Cu bimetal.

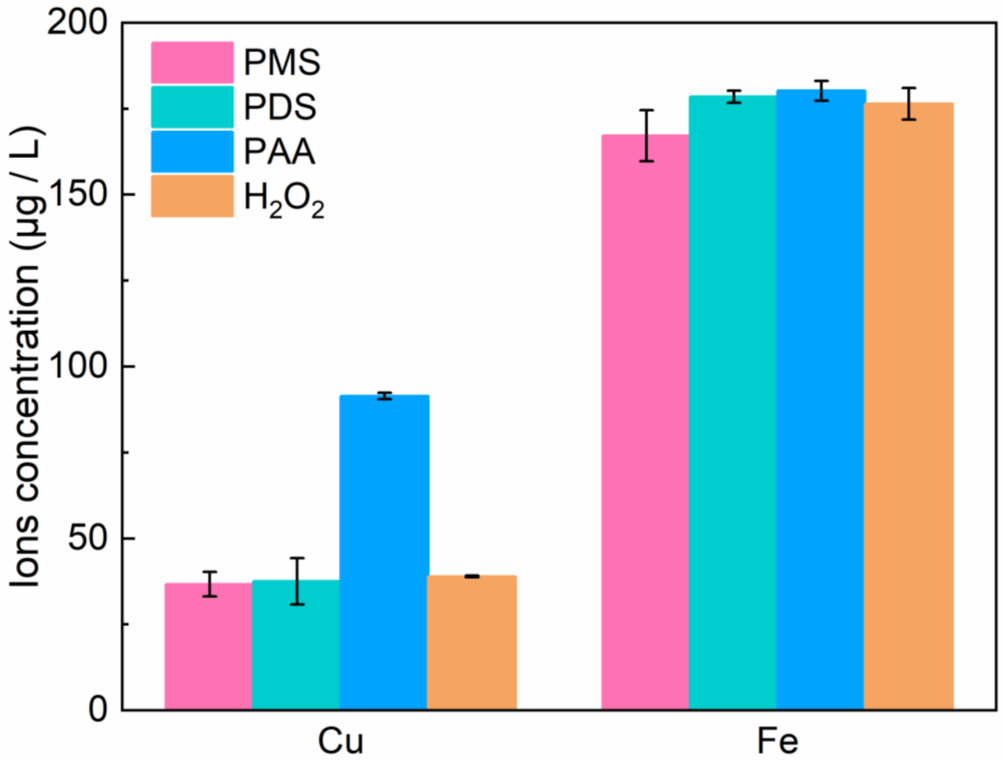


**Figure S7.** Dissolution of iron and copper ions in different systems.

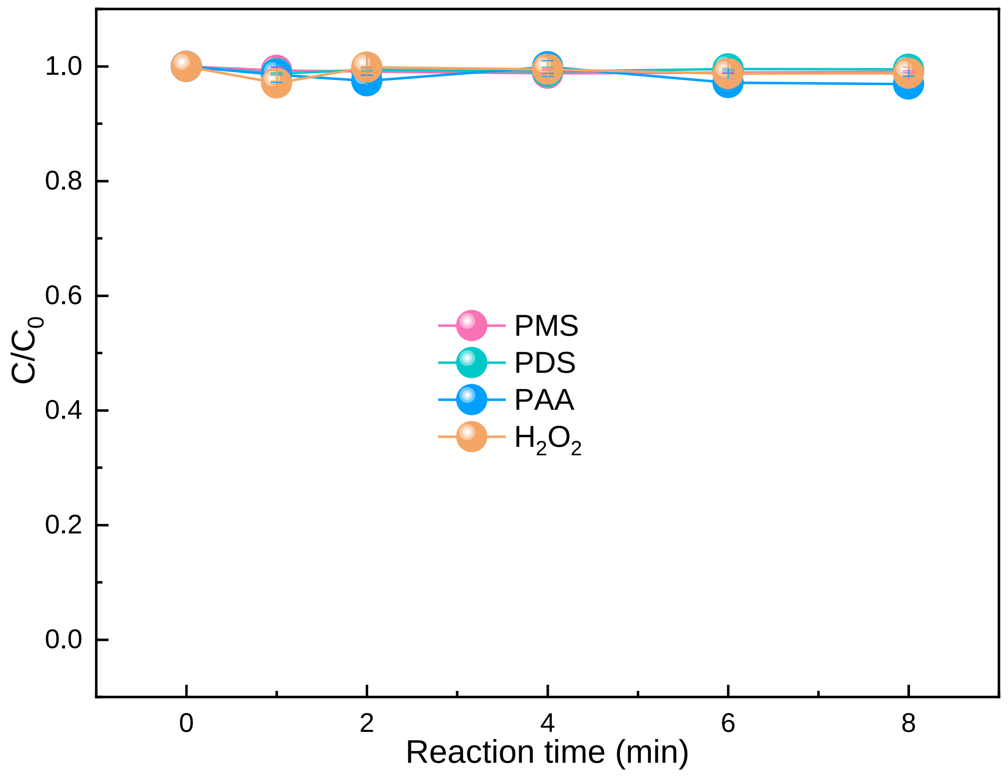


**Figure S8.** Cu ion homogeneous phase experiment.

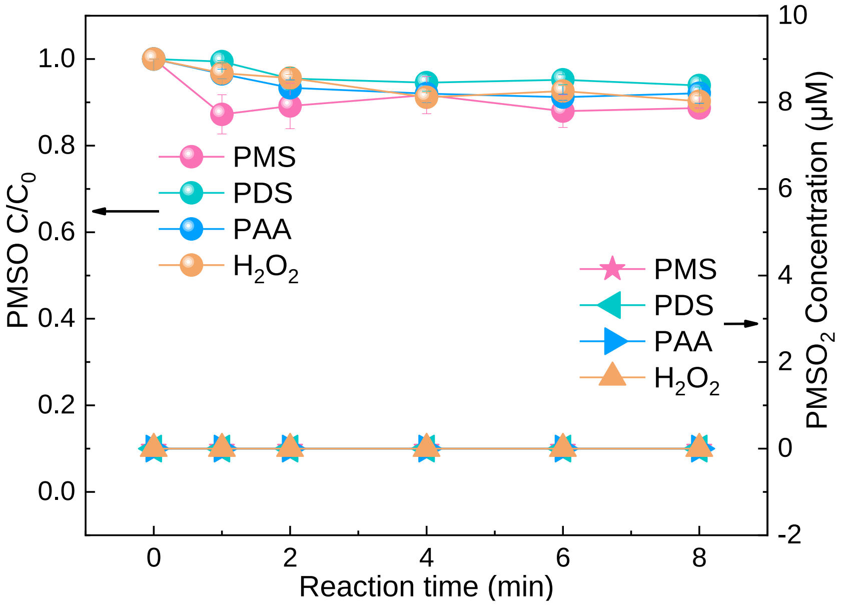


**Figure S9.** PMSO degradation and $PMSO_2$ generation in PMS/PDS/PAA/$H_2O_2$ systems.

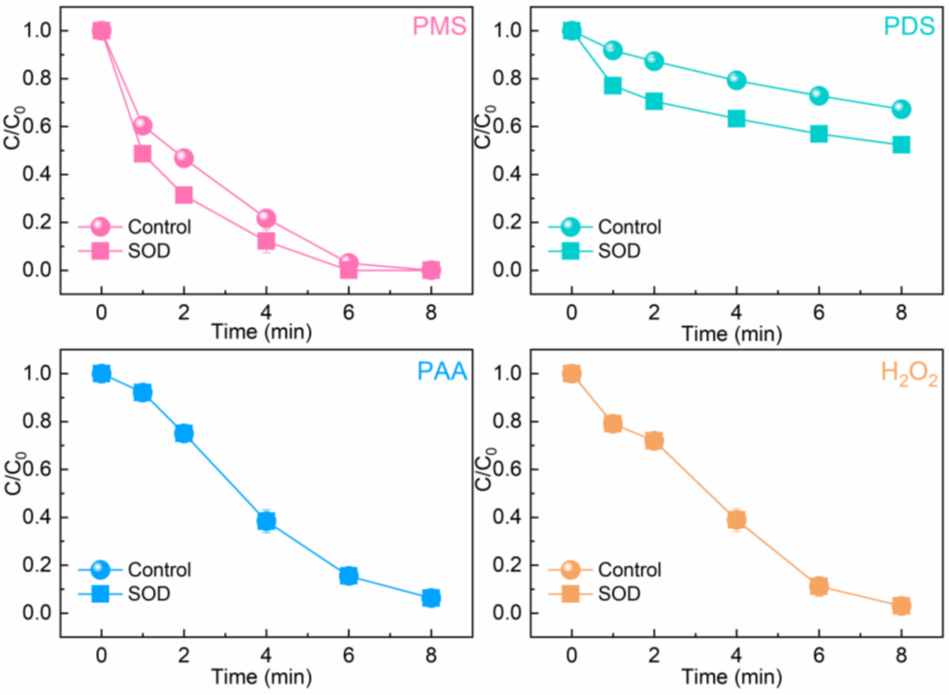


**Figure S10.** Degradation of SMX in four systems after addition of SOD.

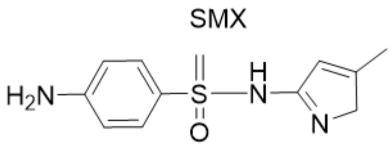


**Figure S11.** Fragment ions identification of SMX.

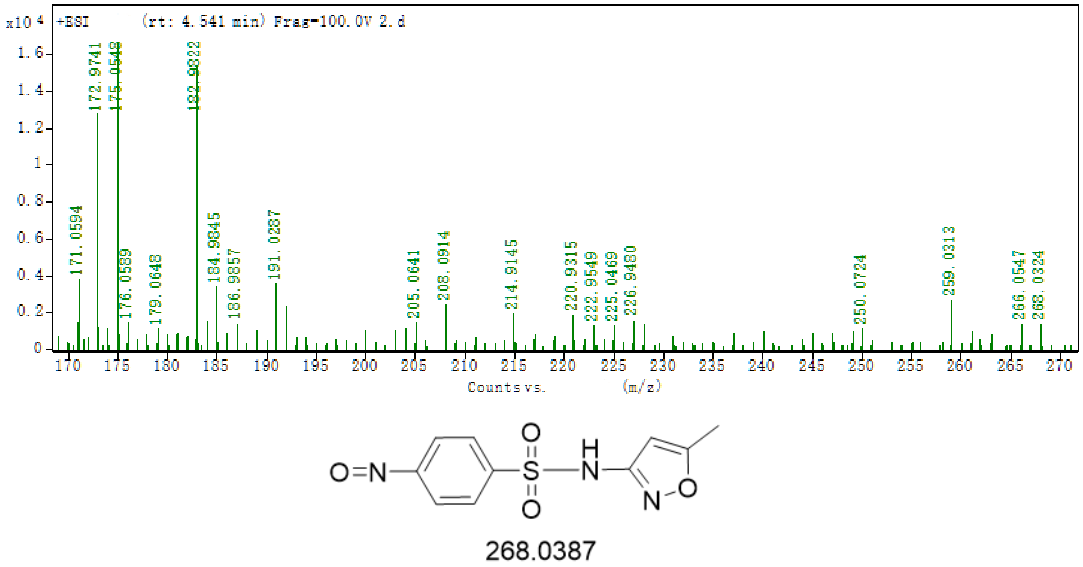


**Figure S12.** Fragment ions identification of P1.

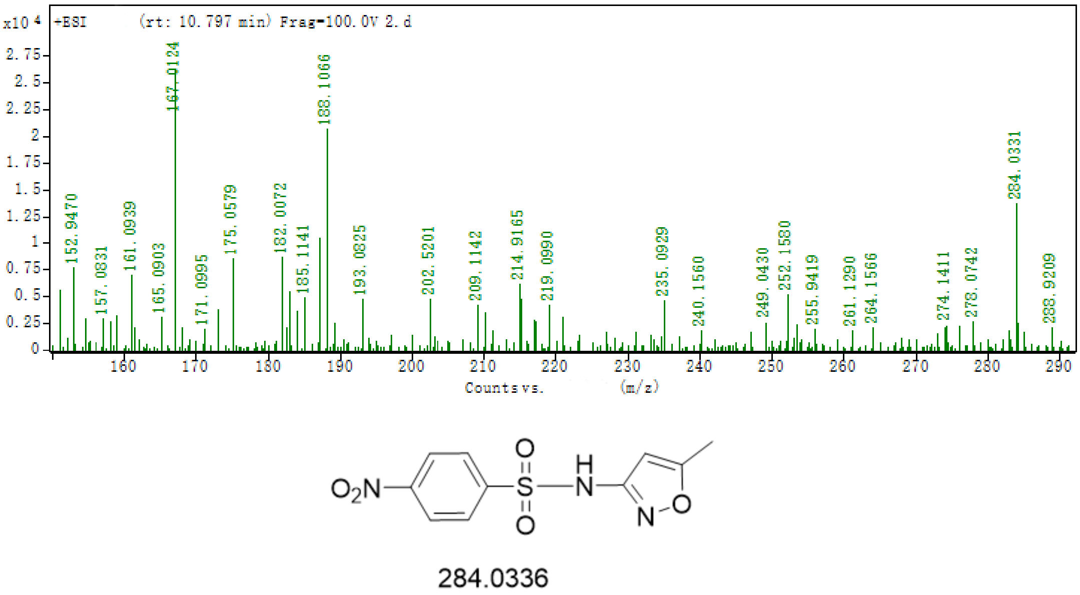


**Figure S13.** Fragment ions identification of P2.

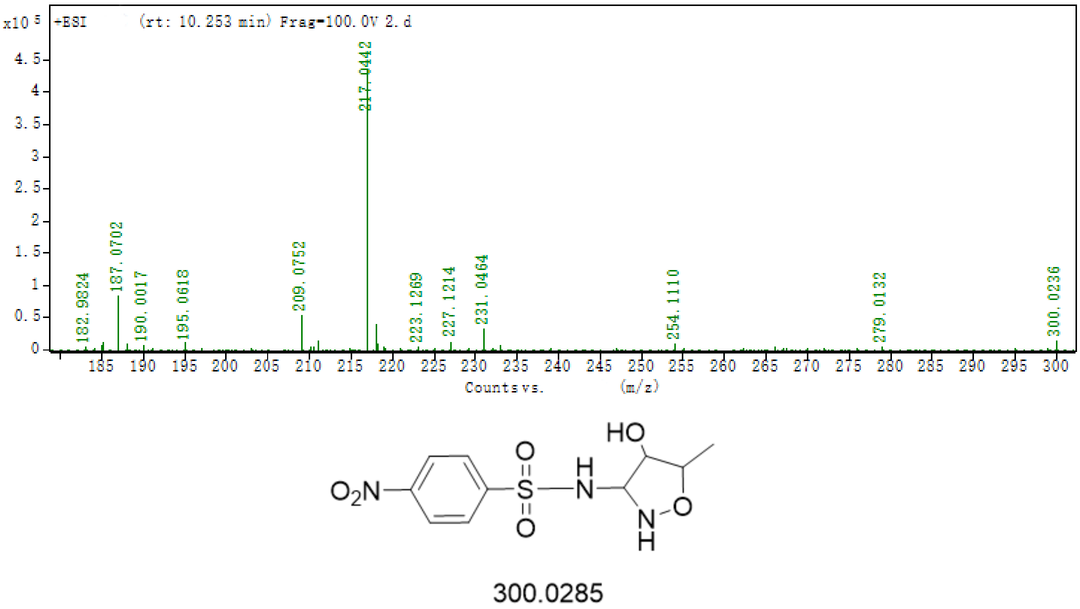


**Figure S14.** Fragment ions identification of P3.

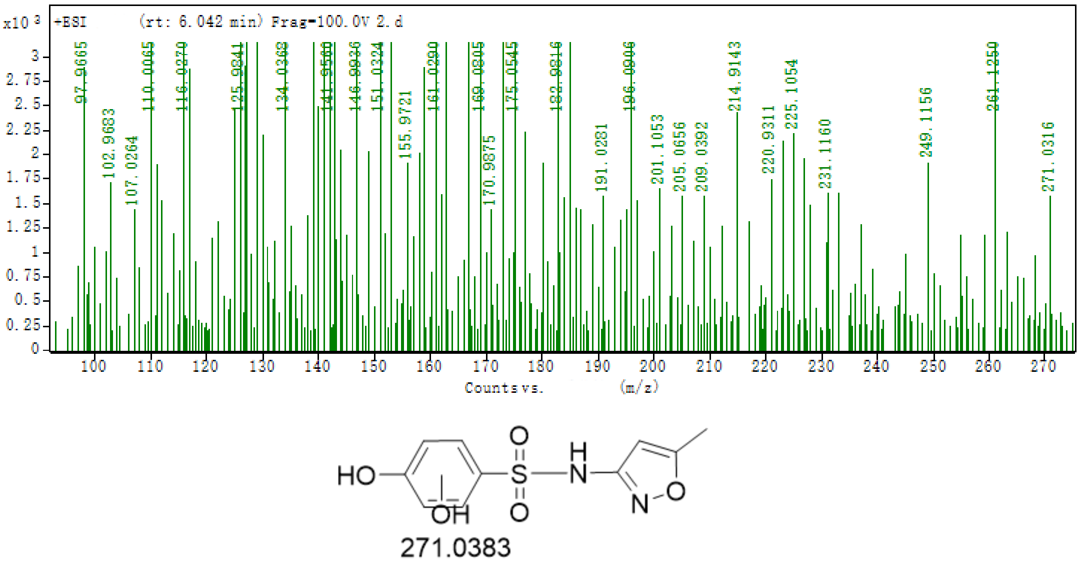


**Figure S15.** Fragment ions identification of P4.

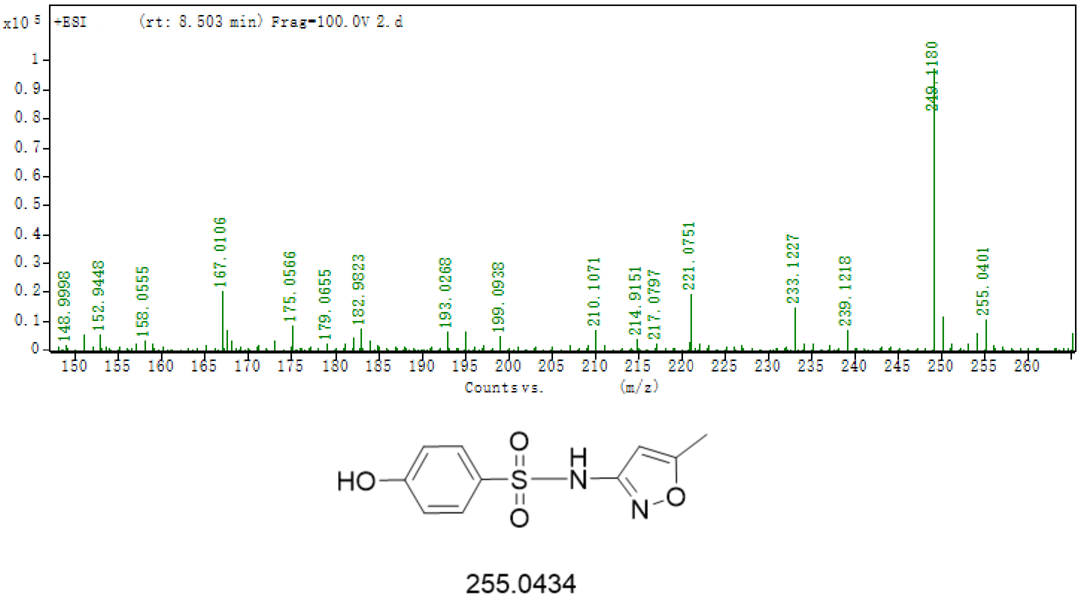


**Figure S16.** Fragment ions identification of P5.

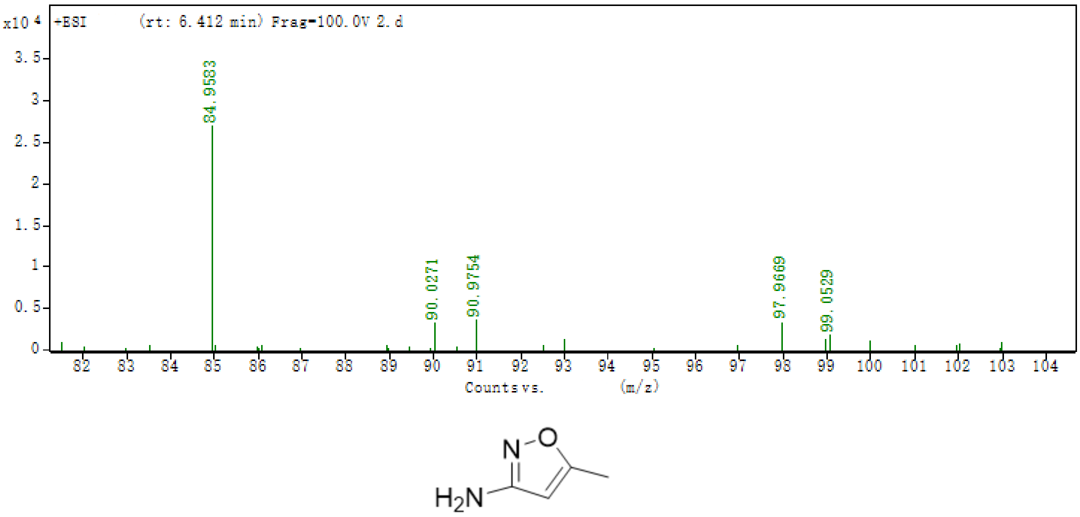


99.0553

**Figure S17.** Fragment ions identification of P6.

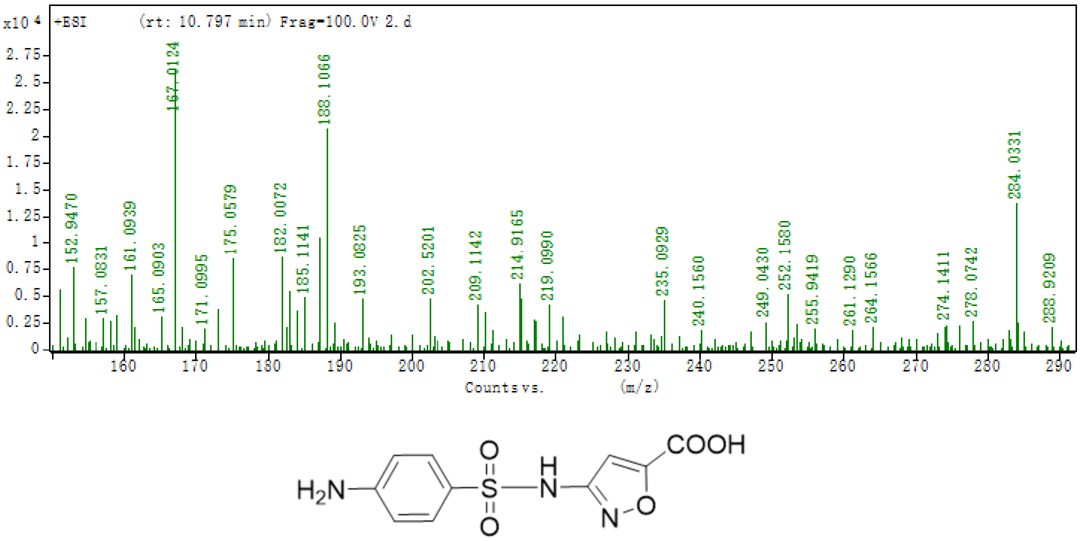


284.0336

**Figure S18.** Fragment ions identification of P7.

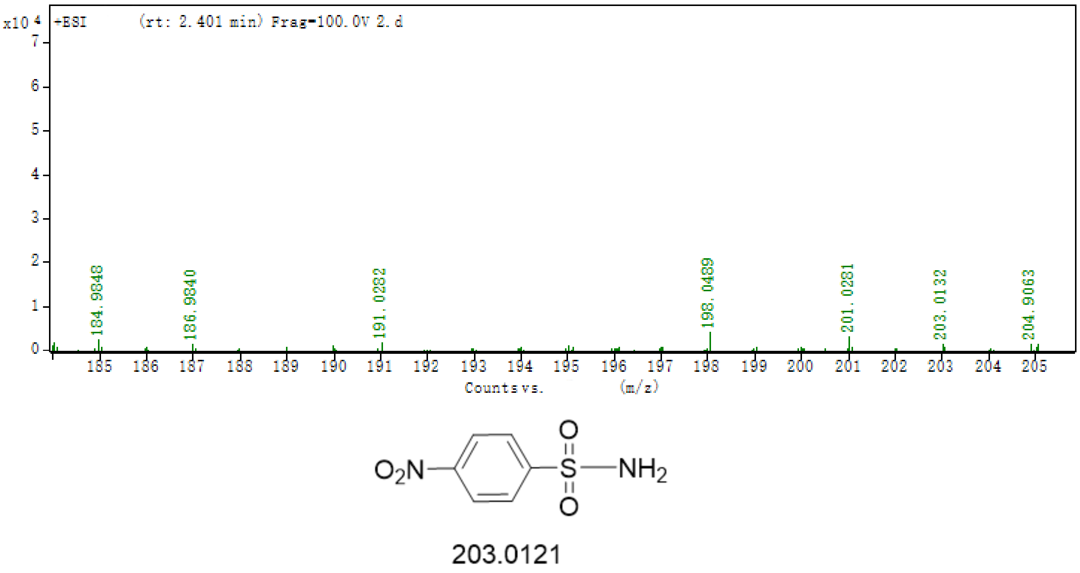


**Figure S19.** Fragment ions identification of P8.

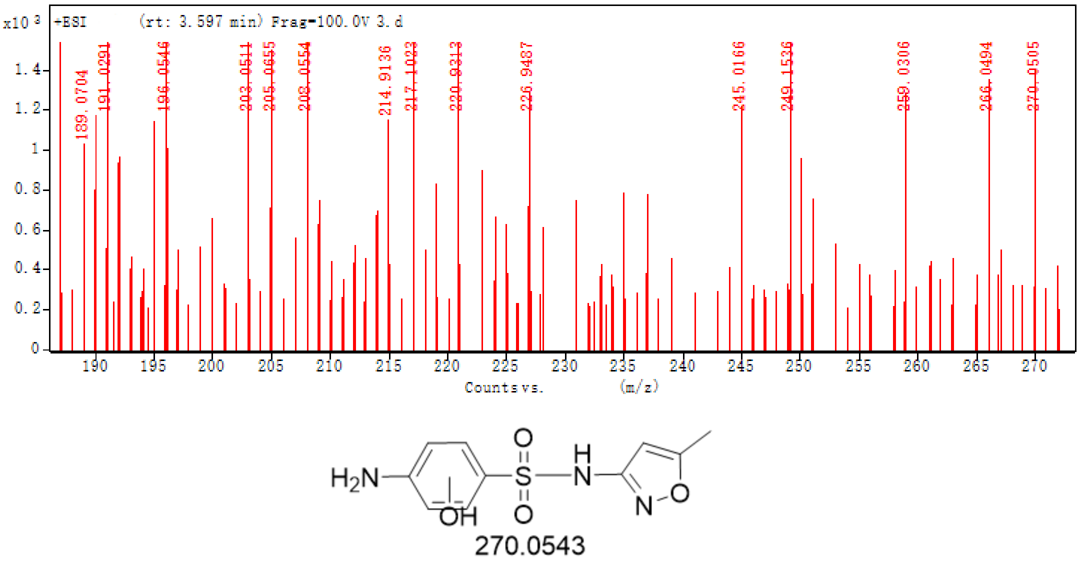


**Figure S20.** Fragment ions identification of P9.

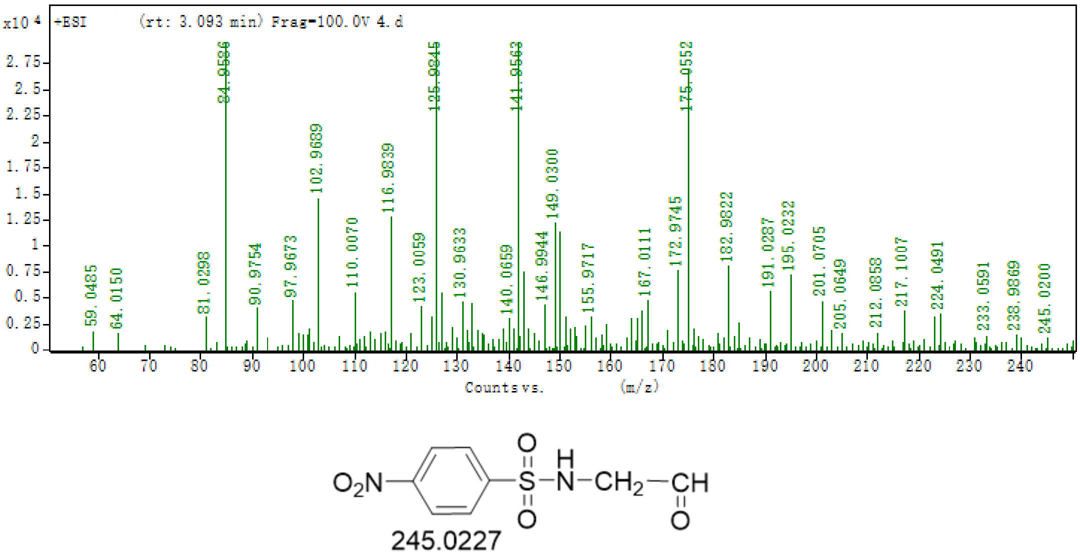


**Figure S21.** Fragment ions identification of P10.

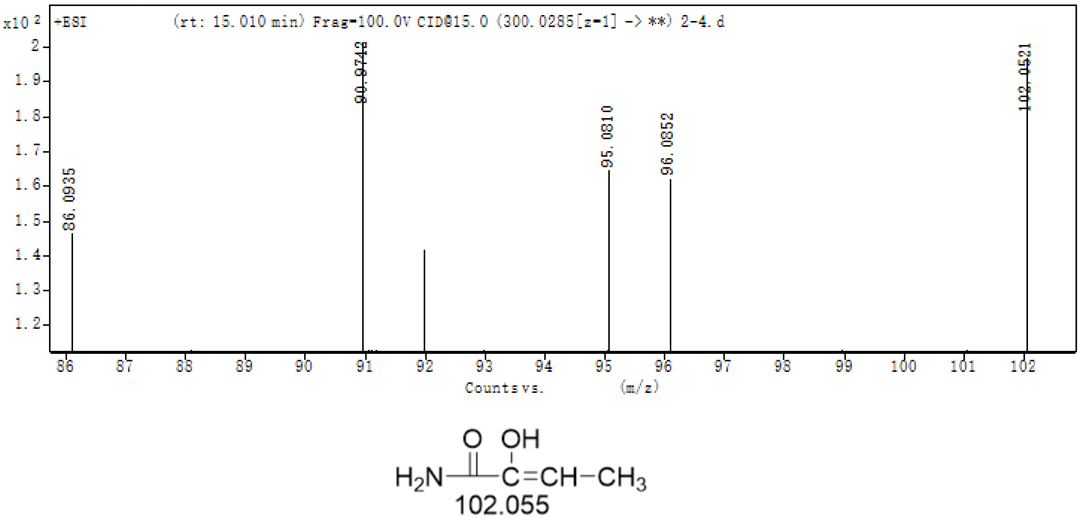


**Figure S22.** Fragment ions identification of P11.

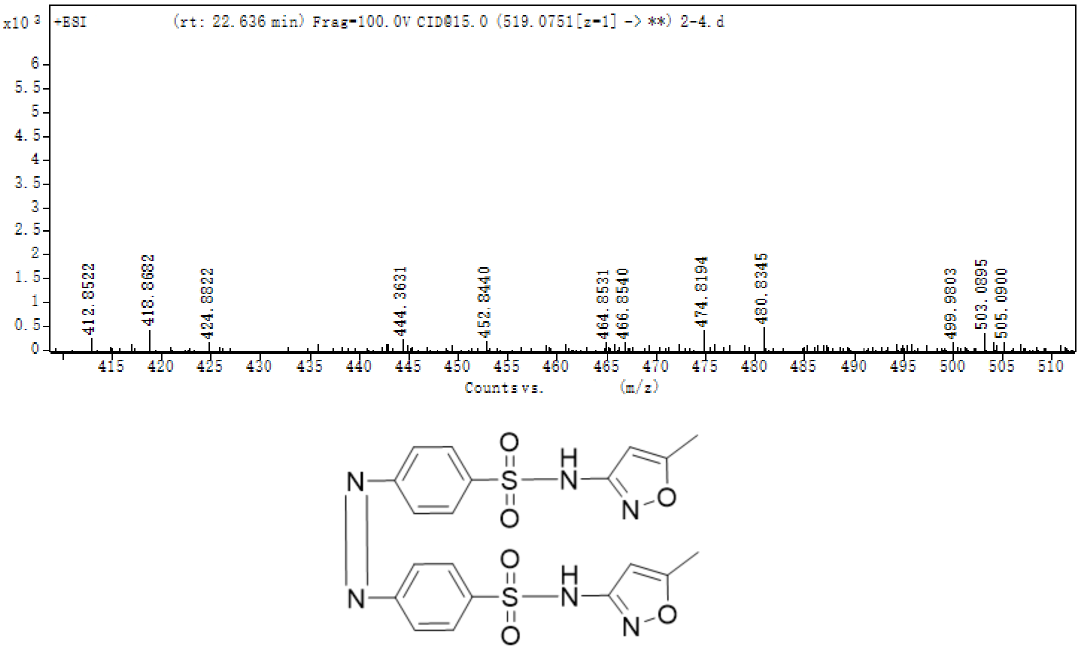


503.0802

**Figure S23.** Fragment ions identification of P12.

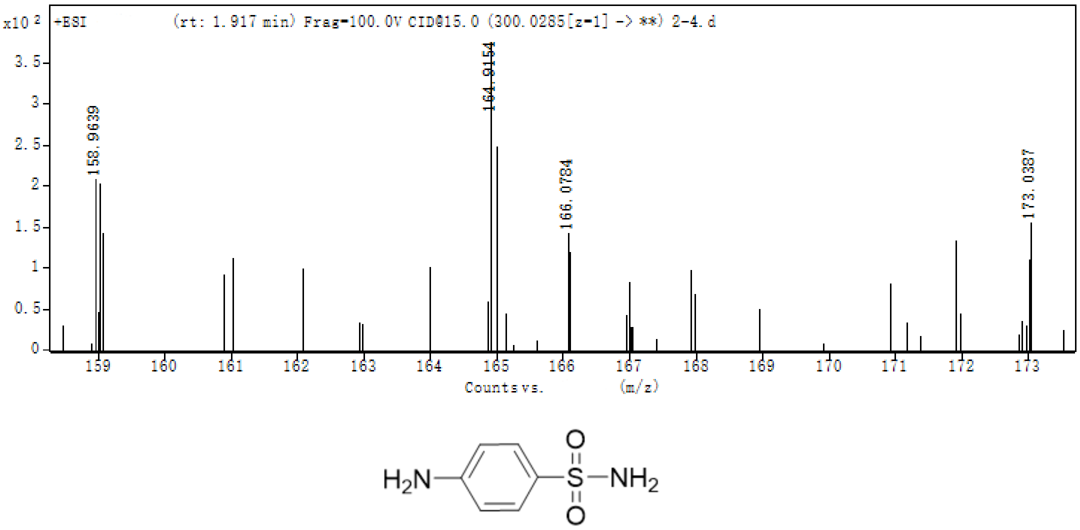


173.0379

**Figure S24.** Fragment ions identification of P13.

**Table S1.** The ICP-OES of Fe-Cu bimetallic.

| Sample number | test element | Sample Elemental ContentW（%) |
| --- | --- | --- |
| 1 | Fe | 93.32% |
| | Cu | 6.62% |

**Table S2.** Transformation intermediates of SMX detected by UPLC-QTOF-MS/MS.

| Product ID | RT(min) | $[M+H]^+$ m/z | Molecular formula | Proposed structure |
|---|---|---|---|---|
| SMX | 7.316 | 254.0594 | $C_{10}H_{11}N_3O_3S$ | 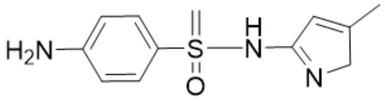 |
| P1 | 4.541 | 268.0387 | $C_{10}H_9N_3O_4S$ | 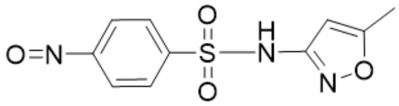 |
| P2 | 10.433 | 284.0336 | $C_{10}H_9N_3O_5S$ | 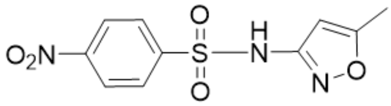 |
| P3 | 10.253 | 300.0285 | $C_{10}H_9N_3O_6S$ | 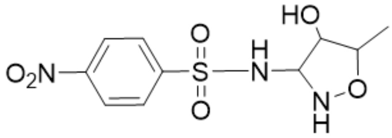 |
| P4 | 6.072 | 271.0383 | $C_{10}H_{10}N_2O_5S$ | 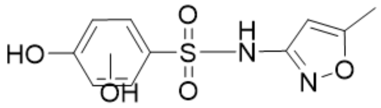 |
| P5 | 8.433 | 255.0434 | $C_{10}H_{10}N_2O_4S$ | 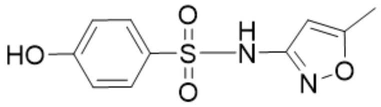 |
| P6 | 6.412 | 99.0553 | $C_4H_6N_2O$ | 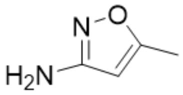 |
| P7 | 10.797 | 284.0336 | $C_{10}H_9N_3O_5S$ | 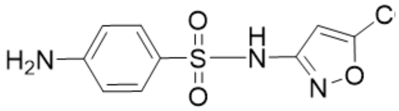 |
| P8 | 2.401 | 203.0121 | $C_6H_6N_2O_4S$ | 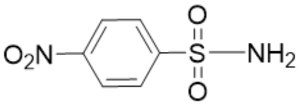 |
| P9 | 3.597 | 270.0543 | $C_{10}H_{11}N_3O_4S$ | 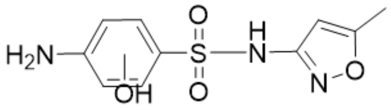 |
| P10 | 3.093 | 245.0227 | $C_8H_8N_2O_5S$ | 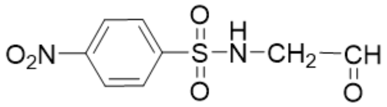 |
| P11 | 15.01 | 102.055 | $C_4H_7NO_2$ | 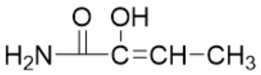 |

| Product ID | RT(min) | $[M+H]^+$ m/z | Molecular formula | Proposed structure |
|---|---|---|---|---|
| P12 | 22.636 | 503.0802 | $C_{20}H_{18}N_6O_6S_2$ | 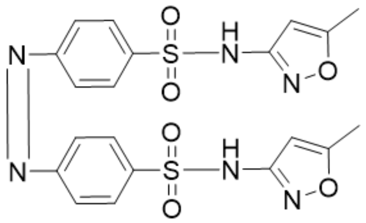 |
| P13 | 1.917 | 173.0379 | $C_6H_6N_2O_4S$ | 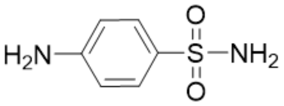 |
